# Evaluating glymphatic pathway function utilizing clinically relevant intrathecal infusion of CSF tracer

**DOI:** 10.1186/1479-5876-11-107

**Published:** 2013-05-01

**Authors:** Lijun Yang, Benjamin T Kress, Harris J Weber, Meenakshisundaram Thiyagarajan, Baozhi Wang, Rashid Deane, Helene Benveniste, Jeffrey J Iliff, Maiken Nedergaard

**Affiliations:** 1 Center for Translational Neuromedicine, Department of Neurosurgery, University of Rochester Medical Center, 601 Elmwood Ave., Rochester, NY, 14642, USA; 2 Department of Neurosurgery, First Hospital of Shijiazhuang, Hebei Medical University, Shijiazhuang, 050031, China; 3 Department of Human Anatomy, Hebei Medical University, Shijiazhuang, 050012, China; 4 Department of Anesthesiology, Stony Brook University, Stony Brook, NY, USA; 5 Department of Anesthesiology and Peri-Operative Medicine, Oregon Health & Science University, Portland, OR, 97239, USA

**Keywords:** Intrathecal infusion, Cerebrospinal fluid (CSF), Perivascular space, Neurodegenerative disease, Alzheimer’s disease, Traumatic brain injury

## Abstract

**Background:**

Neurodegenerative diseases such as Alzheimer’s are associated with the aggregation of endogenous peptides and proteins that contribute to neuronal dysfunction and loss. The glymphatic system, a brain-wide perivascular pathway along which cerebrospinal fluid (CSF) and interstitial fluid (ISF) rapidly exchange, has recently been identified as a key contributor to the clearance of interstitial solutes from the brain, including amyloid β. These findings suggest that measuring changes in glymphatic pathway function may be an important prognostic for evaluating neurodegenerative disease susceptibility or progression. However, no clinically acceptable approach to evaluate glymphatic pathway function in humans has yet been developed.

**Methods:**

Time-sequenced ex vivo fluorescence imaging of coronal rat and mouse brain slices was performed at 30–180 min following intrathecal infusion of CSF tracer (Texas Red- dextran-3, MW 3 kD; FITC- dextran-500, MW 500 kD) into the cisterna magna or lumbar spine. Tracer influx into different brain regions (cortex, white matter, subcortical structures, and hippocampus) in rat was quantified to map the movement of CSF tracer following infusion along both routes, and to determine whether glymphatic pathway function could be evaluated after lumbar intrathecal infusion.

**Results:**

Following lumbar intrathecal infusions, small molecular weight TR-d3 entered the brain along perivascular pathways and exchanged broadly with the brain ISF, consistent with the initial characterization of the glymphatic pathway in mice. Large molecular weight FITC-d500 remained confined to the perivascular spaces. Lumbar intrathecal infusions exhibited a reduced and delayed peak parenchymal fluorescence intensity compared to intracisternal infusions.

**Conclusion:**

Lumbar intrathecal contrast delivery is a clinically useful approach that could be used in conjunction with dynamic contrast enhanced MRI nuclear imaging to assess glymphatic pathway function in humans.

## Background

Neurodegenerative disorders such as Alzheimer’s disease (AD) are characterized by progressive loss of neurons, sensory and motor impairments, and severe cognitive decline [1]. Extracellular depositions of amyloid β (Aβ) peptides and accumulation of hyperphosphorylated tau in neurofibrillary tangles are believed to contribute to the pathophysiology of AD [2-4], while analogous misfolded proteins aggregate in other neurodegenerative diseases, including Huntington’s disease and amyotrophic lateral sclerosis (ALS) [5-7]. In the case of AD, it is widely believed that amyloid deposition results from an age-related failure of soluble Aβ clearance from the brain [8]. Although neuroimaging approaches are now widely available to detect the deposition of amyloid plaques and the static evolution of plaque burden [9], no approach yet exists that allows Aβ clearance efficiency to be directly measured in real time. The ability to detect changes in the efficiency of Aβ clearance would be a significant advance in evaluating susceptibility to and progression of AD in addition to potentially opening up a totally novel therapeutic approach to treatment.

The cerebrospinal fluid (CSF) circulation is widely regarded as a major sink for the clearance of interstitial fluid (ISF) and its solutes from the brain. As CSF is reabsorbed across arachnoid granulations, along cranial and spinal nerve sheaths, or along the brain microvasculature, solutes are cleared from the cranial cavity [10]–[14]. In a recent study we reported that a large proportion of subarachnoid cerebrospinal fluid (CSF) recirculates through the brain parenchyma along perivascular spaces, exchanging with brain interstitial fluid (ISF) before being cleared via peri-venous pathways [15,16]. The continuous circulation of CSF along this pathway facilitates the clearance of extracellular solutes, including soluble Aβ, from the brain. We termed this brain-wide pathway the ‘glymphatic system’, based upon the critical role that astroglial water transport through the astrocytic aquaporin-4 water channel plays in facilitating CSF-ISF exchange and solute clearance [16]. One implication of these findings is that changes in glymphatic pathway function may contribute to the failure of Aβ clearance in the pre-clinical stages of AD, while a method to measure glymphatic pathway function in clinical populations might allow AD disease susceptibility and progression to be evaluated.

Our initial characterization of the glymphatic system utilized in vivo two-photon microscopy and ex vivo fluorescence imaging of intracisternally infused CSF tracers to map the brain-wide pathway and to quantify the efficiency of solute clearance in mice. These approaches are not appropriate for clinical application, given the optical limitations of fluorescence-based imaging and the complications that are associated with intracisternal infusions in humans [17]. In the clinical setting, dynamic nuclear imaging modalities such as magnetic resonance imaging (MRI) are routinely used to monitor CSF flux in the diagnosis of spontaneous intracranial hypotension (SIH) and post-traumatic CSF rhinnorrhea or otorrhea [18,19]. These approaches permit time-sequenced three-dimensional (3D) representation of the brain with high spatial and temporal resolution of tracer distribution. In a follow-up pre-clinical study we have successfully utilized dynamic contrast-enhanced MRI after intrathecal infusion of gadolinium-based contrast agent into the cisterna magna to measure glymphatic pathway function in rats [20,21].

In contrast to intracisternal infusions, lumbar intrathecal injections of radio-tracer are presently used along with computer-tomography/mylography and digital subtraction myelography to diagnose dural leaks associated with SIH, pseudomeningocele, and superficial siderosis [22,23], as well as the integrity of the spinal cord in the setting of injury or tumor [24,25]. Lumbar intrathecal injections are additionally used in everyday practice for the delivery of local anesthetics [26], opioids and other analgesics, and would thus provide an ideal delivery route for contrast agents that could then be used in conjunction with dynamic contrast-enhanced MRI to evaluate glymphatic clearance in humans.

In the present pre-clinical study, we extend these recent findings to evaluate whether perivascular CSF-ISF exchange within the brain can be evaluated after lumbar intrathecal CSF tracer infusion. We compare the influx kinetics and parenchymal distribution of these tracers with those corresponding to intracisternal CSF tracer infusion described previously [16,20]. Our data demonstrate that CSF tracer infused at the lumbar spine enters the brain through perivascular spaces and exchanges with the ISF in a manner consistent with our previous characterization of the glymphatic pathway, suggesting that the lumbar intrathecal infusion is a clinically viable contrast delivery route to asses glymphatic function in humans.

## Methods

### Surgical preparation

Female Sprague–Dawley rats (200–230 g; Charles River Labs, USA) and mixed sex C57BL/6 mice were used in all experiments. The animals were housed under standard laboratory conditions, with access to food and water, *ad libitum*. All experiments were approved by the University Committee on Animal Resources of the University of Rochester and carried out according to guidelines from the National Institutes of Health. Animals were initially induced with isofluorane (3%), and then anesthetized with sodium pentobarbital (50 mg/kg i.p.). Supplemental sodium pentobarbital (25 mg/kg) was given as necessary. For intracisternal infusions, anesthetized rats were fixed in a stereotaxic frame, the atlanto-occipital membrane was surgically exposed followed by a durotomy, and a 30GA needle was inserted into the cisterna magna. For lumbar infusions, the lumbar spinal column was exposed, an L_2-3_ laminectomy was performed, and 30GA needle was inserted into the subarachnoid space.

### Tracer infusion

In the present study, 2 different fluorescent tracers were used: large molecular weight fixable fluorescein isothiocyanate (FITC)-conjugated dextran (500 kD, FITC-d500; Invitrogen) and small molecular weight fixable Texas Red-conjugated dextran (3 k, TR-d3; Invitrogen). FITC-d500 and TR-d3 were selected to roughly correspond with the molecular weights of Gd-DTPA (MW ~ 1 kD) and GadoSpin™ (MW 200 kD), which are commonly used MRI contrast agents [27]. Tracers were constituted in isobaric artificial CSF at a concentration of 0.25%. In a companion study [20], we evaluated CSF-ISF exchange after intracisternal infusion of MRI contrast agents and found that two key components of the glymphatic system (i.e. para-arterial CSF tracer influx and size-dependent CSF-ISF exchange) could be visualized with dynamic contrast-enhanced MRI. In order to permit comparison with this prior study [20], in the present study intracisternal and lumbar infusions in rats were conducted in the same manner: an infusion rate of 1.6 μl/min for each of the 30, 60, 120, and 180 min groups. Infusion was stopped at a total volume of 70 μl. At t = 30, 60, or 120 min after the start of infusion, rats were transcardially perfused with ice-cold heparinized PBS, followed by 4% paraformaldehyde (PFA). Brains were removed, post-fixed overnight in 4% PFA at 4°C, and 50 μm coronal slices were cut on a vibratome (Leica) and mounted with PROLONG Anti-Fade Gold with DAPI (Invitrogen). For studies conducted in mice, TR-d3 was infused via the cisterna magna at a rate of 1.6 μl/min for 6.25 min (total volume = 10 μl). This infusion volume was the same as was used previously to define perivascular CSF-ISF exchange by in vivo 2-photon microscopy [16].

In a separate group of animals, we evaluated the effect of tracer infusion upon intracranial pressure (ICP). A 22GA ICP cannula was inserted stereotactically into the right lateral ventrical and the animal allowed to equilibrate for 30 min. In the first animals, we sequentially increased intracisternal CSF tracer infusion rates from 1.6 to 3.2 to 6.4 μl/min to define the rate that would not evoke an acute elevation of ICP. In 2 additional groups of animals (n = 4 each), we measured ICP during 60 min infusions of CSF tracer via the cisterna magna and the lumbar spine.

### Immunofluorescence

Free floating immunofluorescent labeling was conducted on a separate set of 50 μm slices. For intracisternal infusion, 8 slices from each animal (n = 3 to 4 rats per time point) from the 60 min time point were used. For lumbar infusion, 8 slices from each animal from the 120 min time point were used. These time points were chosen based on pilot data, because they corresponded with peak CSF tracer influx via the respective infusion routes. Slices were blocked in 3% normal donkey serum (Jackson Immunoresearch) for 1.5 hrs at room temperature, incubated in mouse anti-glial fibrillary acidic protein (GFAP, an astrocytic marker; 1:1000, Millipore) or Biotinylated Griffonia (Brandeiraea) Simplicifolia Lectin I Isolectin B4 (Ib4, a vascular endothelial marker; 1:100, Vector Laboratories) overnight at 4°C. Secondary detection was conducted for mouse-anti GFAP using Cy5-conjugated donkey anti-mouse secondary antibody (1:500, Jackson Immunoresearch) and for Ib4 using Cy5-conjugated streptavidin (1:250, Jackson Immunoresearch). Slices were incubated for 2 hrs at room temperature in the secondary antibody solution. After washing, all slices were mounted with PROLONG Antifade Gold with DAPI (Invitrogen).

### Fluorescence imaging

Tracer movement into the brain was measured by conventional fluorescence microscopy. Whole slice 3-channel montages were generated at 4x magnification for 8 slices per animal using the Virtual Slice module of the Microlucida software (Microbrighfield), on an upright fluorescence microscope (Olympus) equipped with a motorized stage. This included separate DAPI, green (for FITC) and red (for Texas Red) emission channels. The gain and exposure settings were determined based on un-infused control slices and held constant throughout all imaging sessions. Slice fluorescence (fluorescent tracer accumulation) was quantified using ImageJ software (NIH), as described previously [16]. Anatomical brain regions of interest (ROIs) were generated based upon the DAPI emission channel for the cortex, white matter (including corpus callosum, internal and external capsule), subcortical structures (striatum, thalamus, hypothalamus) and hippocampus. The background fluorescence for each channel was uniformly subtracted and the mean pixel intensity for each region was measured. Distribution of large and small molecular weight CSF tracers throughout the brain parenchyma was evaluated under high power by laser scanning confocal microscopy (Olympus). Immunofluorescently-labeled slices were imaged at 40X objective power.

### Statistical analysis

All values are expressed as mean ± SEM. Intensities were compared at each time point and across infusion routes by 2-way analysis of variance (ANOVA) with Bonferroni’s post-hoc test to evaluate differences at individual time points (Graphpad Prism software). A ‘P’ value < 0.05 was considered significant.

## Results and discussion

In two recent studies, 2-photon in vivo imaging in mice and dynamic contrast-enhanced MRI in rats were used to demonstrate the existence of a brain-wide perivascular route, termed the ‘glymphatic pathway’, that permits CSF to exchange with the brain ISF [16,20]. CSF-ISF exchange along these perivascular pathways was supported by astroglial aquaporin-4 water channels and the movement of fluid through this pathway facilitated the clearance of interstitial solutes, including soluble Aβ, from the brain. The purpose of the present study was to assess whether a clinically relevant lumbar intrathecal route of CSF tracer delivery could be used to visualize perivascular CSF-ISF exchange in the brain. In contrast to our initial study, which was performed on mice, the current study characterized glymphatic flow in the adult Sprague Dawley rat, which expands our understanding of glymphatic function into a new species, while also moving to a model system that makes it easier to perform lumbar intrathecal infusions. Intracisternal CSF tracer infusions were carried out in a parallel group of animals to provide a direct comparison to the infusion route used previously [16,20].

### Effect of intrathecal lumbar and cisternal tracer infusion upon intracranial pressure

**Figure 1:**
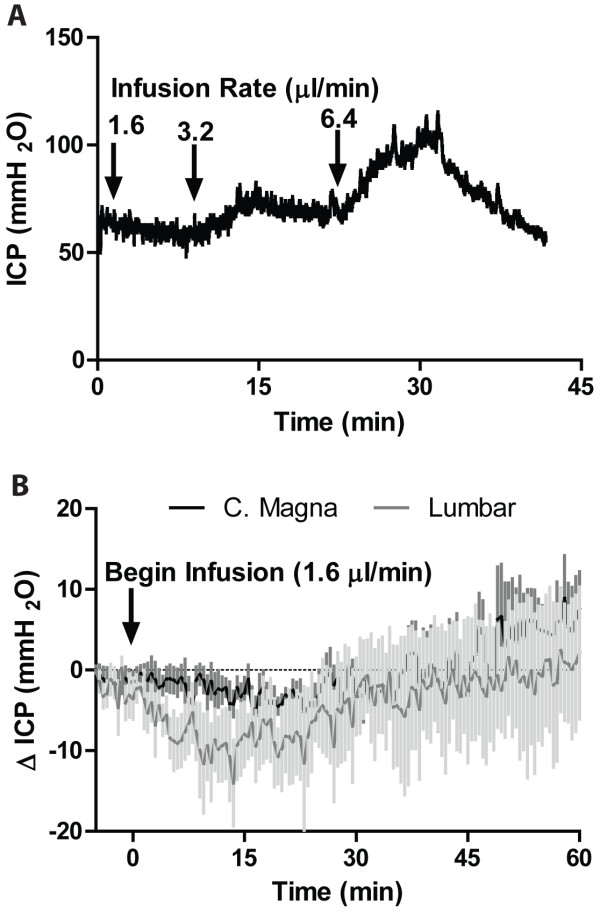
**Defining the effect of intrathecal tracer infusion on intracranial pressure (ICP).** Ventricular ICP was monitored continuously during infusion of CSF tracer Texas Red-conjugated dextran (TR-d3, MW 3 kD) in rats. (**A**) Intracisternal TR-d3 infusion at 1.6 ul/min did not appreciatively alter ICP, whereas increasing the infusion rate to 3.2 and 6.4 ul/min significantly elevated ICP. (**B**) Intracisternal and lumbar infusion of TR-d3 did at 1.6 μl/min for 60min did not significantly alter ICP (n = 4 per group).

Although a bolus injection would be the easiest to translate to the clinical setting, we found in pilot studies that the intracranial hypertension resulting from a bolus injection of fluorescent tracer or contrast agent at the cisterna magna was fatal. This limitation would likely not be a factor in larger species, such as non-human primates or humans. When ventricular ICP was measured in rats during intracisternal TR-d3 infusion, we observed that infusing at a rate of 1.6 μl/min did not appreciatively alter ICP (Figure 1A). When infusion rate was increased to 3.2 and 6.4 μl/min, a progressive elevation in ICP was noted (Figure 1A). Based upon these findings, we conducted lumbar and intracisternal tracer infusion at 1.6 μl/min for 60 min and measured the effect upon ICP. We observed that in the rat under the present anesthetic conditions, a tracer infusion rate of 1.6 ul/min did not significantly alter ICP (Figure 1B).

### Imaging CSF-ISF exchange in mouse and rat by intracisternal tracer infusion

**Figure 2:**
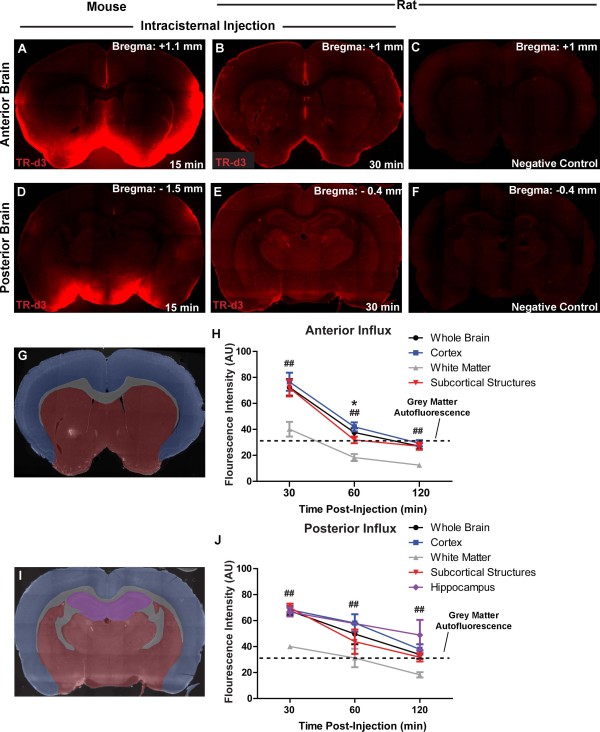
**Evaluating intracisternal CSF tracer influx and clearance in mouse and rat.** Representative anterior (**A**-**B**) and posterior (**D**-**E**) coronal slices from mouse (**A**, **D**) and rat (**B**, **E**) brains following intracisternal infusion of Texas Red-conjugated dextran (TR-d3, MW 3kD; t = 30min post-infusion) show similar tracer distributions between species. (**C**, **F**) Un-infused rat brain slices exhibit little tissue autofluorescence. (**G**-**J**) Tissue fluorescence was evaluated in different brain regions: cortex (blue), white matter (grey), hippocampus (magenta), and subcortical structures (red) of the anterior (**G**-**H**) and posterior (**I-J**) brain. (**H**, **J**) Quantification of mean fluorescence intensity within each region (*P < 0.05 cortex vs. subcortical structures; ^##^P < 0.01 cortex vs. white matter structures; 2-way ANOVA; n = 3–4 per time point). Dashed line indicates average gray matter tissue autofluorescence level.

We next characterized the movement of intrathecal CSF fluorescent tracer (TR-d3) into the rat brain parenchyma after infusion via the cisterna magna. Figure 2A-B, D-E shows representative images from mouse and rat brain slices taken 1.1 and 1.0 mm, respectively, anterior and 1.5 and 0.4 mm, respectively, posterior to Bregma, that were fixed 30 min after the start of tracer infusion (the time point of peak fluorescence intensity). Under the same imaging conditions, un-infused brain tissue exhibited only limited fluorescence (Figure 2C,F). Consistent with our prior study in mice [16], we observed that intracisternally-infused TR-d3 moved rapidly into the rat brain parenchyma, both across the pial surface and along perivascular pathways (Figure 2B,E) and exchanged broadly with the brain interstitium. To quantify tracer movement into different brain regions, we defined four anterior (Figure 2G-H) and posterior (Figure 2I-J) regions of interest including the cortex, white matter, subcortical structures, and hippocampus (Figure 2G,I show the regions of interest). Analysis of TR-d3 fluorescence either within the whole ex vivo slice or within the individual regions revealed that the influx of intracisternally-infused CSF tracer peaked around 30 min post-infusion, then began to decline at later time points as the tracer was cleared from the brain tissue (Figure 2H,J; n = 3–4 animals per time point).

Analysis of tracer clearance between the four different anatomical regions suggested that tracer clearance from subcortical structures was faster than from the cortex (Figure 2H,J; *P < 0.05 cortex vs. subcortical, 2-way ANOVA). This is consistent with our prior observation that subcortical regions enjoy the largest influxes of subarachnoid CSF along large caliber penetrating arteries from the ventral brain surface [16]. Furthermore, these regions are most proximate to the para-venous clearance pathways that drain medially along the internal cerebral veins. When anterior versus posterior tracer fluxes were compared, both peak values at 30 min and the rate of clearance between 30–120 min post-infusion were lower in the posterior brain compared to the anterior brain (compare Figure 2H,J). These observations are consistent with MRI findings in the rat in which glymphatic fluxes along anterior penetrating arteries were both faster and greater in magnitude than those that followed branches of mediolateral arteries such as the middle cerebral artery [20]. In both the anterior and posterior brain, TR-d3 fluorescence intensity in the white matter was significantly lower than the cortex at all time points (Figure 2H,J; ^##^P < 0.01 vs. cortex, 2-way ANOVA), while the rate of tracer clearance from the white matter did not appear to consistently differ from other brain regions. The shift in white matter fluorescence intensity likely stems from lower levels of tissue autofluorescence of white matter compared to gray matter. Alternatively, this may reflect the lower levels of CSF tracer penetration into the subcortical white matter observed in our prior study [16].

### Imaging rat CSF-ISF exchange following lumbar intrathecal infusions

**Figure 3:**
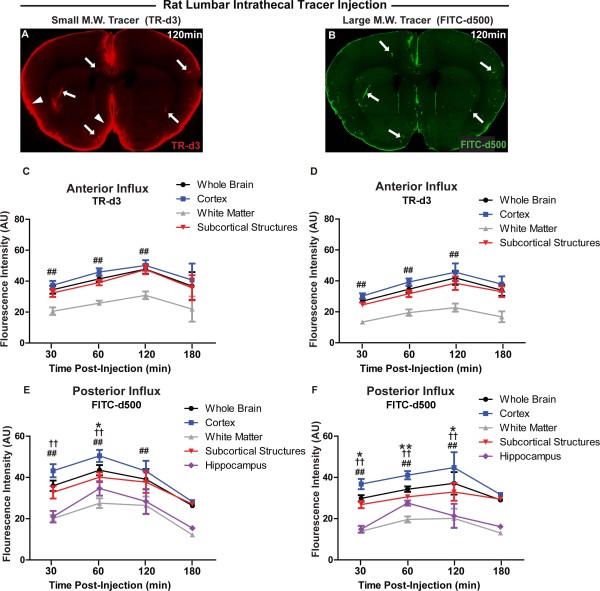
**Effect of molecular weight on tracer influx into the brain after lumbar intrathecal infusion.** (**A**-**B**) Coronal brain slices show penetration of large molecular weight FITC-conjugated dextran (FITC-d500, MW 500kD) and small molecular weight Texas Red-conjugated dextran (TR-d3, MW 3kD) 120 min after lumbar intrathecal co-infusion. FITC-d500 is largely confined to perivascular spaces (**B**, arrows), while TR-d3 moves readily though the brain parenchyma from perivascular spaces (**A**, arrows) or from the pial surface (arrowheads). (**C**-**F**) Quantification of fluorescent tracer influx into anterior (**C**-**D**) and posterior (**E**-**F**) brain after lumbar intrathecal infusion, anatomically subdivided into cortex, white matter, subcortical structures, and hippocampus.(*P < 0.05, *P < 0.01 cortex vs. subcortical structures; ^##^P < 0.01 cortex vs. white matter; ^†^P < 0.05, ^††^P < 0.01 cortex vs. hippocampus; 2-way ANOVA; n = 3-4 per time point).

Compared to intracisternally-infused TR-d3 (Figure 2H,J), the influx of CSF tracer delivered by the lumbar route was significantly delayed, peaking in the posterior brain 60 min after infusion, and in the anterior brain 120 min after infusion (compare Figure 2H,J to Figure 3C,E). This delay in CSF tracer influx kinetics is likely attributable to two factors. First, the distance from the site of lumbar infusion to the cisterna magna is ~32 mm. Second, the prevailing direction of CSF bulk flow within the spinal subarachnoid is rostral-to-caudal [28], suggesting that during infusion, CSF tracer must traverse the intervening distance against the bulk CSF flow generated from CSF secretion in the cerebral ventricles. In addition to the delay in fluorescent CSF tracer influx, the overall magnitude of tracer influx into the brain was markedly reduced after lumbar infusion compared to intracisternal infusion. The density of the infusate likely did not play a significant role in directing tracer distribution or kinetics, as the infusate consisting of either of the two tracers was determined to be isobaric to the CSF. This difference is most likely the result of CSF reabsorption that occurs along the spinal column via the peripheral nerve roots [29], or perhaps subarachnoid CSF diversion along spinal cord perivascular routes and into the central canal, as has been reported following lumbar intrathecal infusion of horseradish peroxidase [30]. As CSF tracer is infused into the lumbar subarachnoid space and moves rostrally towards the brain, a portion will be deposited along each vertebral segment along the natural CSF clearance pathway, resulting a reduced delivery of tracer to the distant cisternal spaces surrounding the brain which form the entrance to the brain-wide glymphatic system [16,20]. Despite these differences in influx magnitude and kinetics, tracer distribution after lumbar and intracisternal infusion followed a similar pattern and encompassed the entire brain parenchyma (compare Figure 2B,E and Figure 3A). These findings demonstrate that fluorescent intrathecal tracer influx into and through the brain parenchyma can be readily visualized after infusion via a lumbar route.

### Rat lumbar intrathecal tracer influx is independent of molecular weight

CSF moves through the ventricular and subarachnoid compartment through the process of bulk flow [13,31,32]. Because under bulk flow, the movement of the solvent is typically more rapid than the movement of the solute by passive diffusion, bulk flow-dependent movement is largely independent of molecular weight [31]. To evaluate whether the rate of intrathecal lumbar tracer influx into the brain parenchyma was dependent upon molecular size, we co-infused large molecular weight fluorescent FITC-d500 (MW 500 kD) and small molecular weight TR-d3 (MW 3 kD). Representative images from this study are shown in Figure 3A-B. When FITC-d500 and TR-d3 movement into the brain parenchyma were quantified within the anterior and posterior cortex, white matter, subcortical structures and hippocampus, no significant differences were observed between either the time course or the magnitude of influx of the small and large molecular weight tracers (Figure 3C-F). This finding is consistent with the movement of fluorescent tracer from the lumbar site of infusion to the brain subarachnoid and perivascular spaces via bulk flow rather than by simple diffusion [31].

### Perivascular pathway of intracthecal CSF tracer influx

In our prior study in mice [16], we noted that while CSF tracers of all sizes moved rapidly into the brain along perivascular spaces, large molecular weight tracers such as FITC-d2000 (MW 2000 kD) became trapped in the perivascular space and could not move freely into and through the brain interstitium. According to one recent study, astrocytic endfoot processes completely cover the cerebral microcirculation; the only routes between the perivascular spaces and the wider brain interstitium being through ~20 nm clefts between overlapping astroglial endfeet [33]. We surmised that the perivascular astrocytic endfeet acted as a sieve to restrict the movement of large solutes from the perivascular spaces into the brain interstitium [16]. This is consistent of with diameters of hydration measured for both 3 kD dextrans (<20 nm) and 500 kD dextrans (>20 nm) [34].

**Figure 4:**
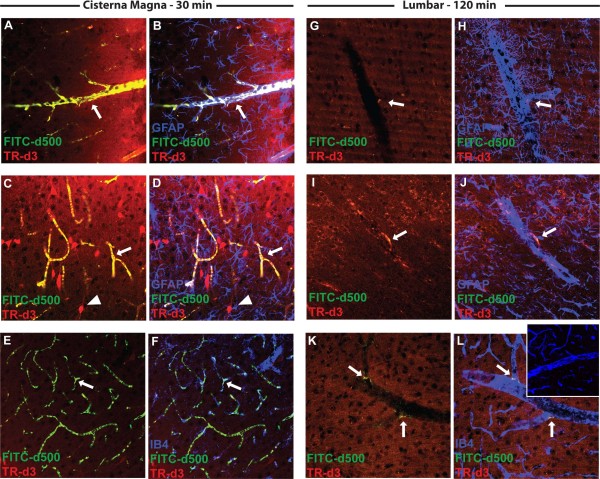
**CSF tracer localization after intracisternal and lumbar intrathecal infusion.** (**A**-**F**) Localization of FITC-conjugated dextran (FITC-d500, MW 500kD) and Texas Red-conjugated dextran (TR-d3, MW 3 kD) 30 min after intracisternal infusion. Large molecular weight FITC-d500 remained restricted to perivascular spaces (arrows) surrounding penetrating arteries (**A**-**B**) and extending to the level of the terminal capillary beds (**C**-**F**, arrows). Small molecular weight TR-d3 moved quickly into the brain interstitium and was taken up by subpopulations of neurons (arrowheads). (**G**-**L**) 120 min after lumbar intrathecal infusion of tracers, large molecular weight FITC-d500 accumulated in perivascular spaces (arrows), but not as uniformly as observed after intracisternal infusion. Small molecular weight TR-d3 moved readily throughout the brain parenchyma. Inset (**L**) depicts IB4 staining and background green and red fluorescence in tissue from animals not injected with CSF tracer. GFAP: glial fibrillary acidic protein (astrocytic marker); IB4: isolectin B4 (vascular endothelial marker).

In the present study, we utilized confocal microscopy to evaluate small (TR-d3) and large (FITC-d500) fluorescent CSF tracer exchange between the perivascular influx pathway and the surrounding brain interstitium. We fixed slices from intracisternally-infused animals and lumbar-infused animals at 30 and 120 min after infusion, respectively. These time points corresponded to the peak influx values observed for each infusion route (Figures 2–3). Following intracisternal infusion, FITC-d500 covered the pial surface and distributed into deeper brain tissue along perivascular pathways, extending to the level of the terminal capillary beds (Figure 4A-F). It did not move appreciably from the perivascular spaces into the surrounding interstitium. When FITC-d500 distribution was evaluated after lumbar infusion, the large molecular weight tracer in a similar manner remained confined to perivascular spaces (Figure 4G-L), however the overall fluorescence intensity was reduced compared to intracisternal infusions. After both intracisternal and lumbar infusion, small molecular weight tracer moved readily into the interstitium surrounding perivascular spaces (Figure 4A-L). Importantly, when tissue was processed and imaged from animals not undergoing CSF tracer injection, background green and red fluorescence was negligible (Figure 4L, inset), indicating that observed tissue fluorescence stemmed from the influx of fluorescent CSF tracer. These data demonstrate that after both intracisternal and lumbar intrathecal infusion in the rat, large molecular weight fluorescent tracers move into the brain along perivascular spaces, but remain confined to this space. Small molecular weight CSF tracers, in contrast, are able to move into and through the brain interstitium.

## Conclusion

In the present pre-clinical study, we report that perivascular CSF-ISF exchange within the rat brain can be evaluated following infusion of CSF tracer via a lumbar, in addition to an inctracisternal, route. In order to translate the initial characterization of the glymphatic pathway to a clinical setting, it is necessary to use a clinically relevant methodology for CSF tracer and contrast infusion as well as imaging modality for evaluation of glymphatic pathway function. A recent analysis showed that the glymphatic system can be evaluated in the rats using contrast-enhanced MRI following intracisternal gadolinium-based contrast infusion [20]. Intracisternal infusions, however, are rarely used in a clinical setting due to their high potential for iatrogenic complications, including traumatic tissue injury [17]. In contrast, the lumbar intrathecal delivery route is routinely employed for administration of contrast for CT-myelography [23,25], spinal anesthetics and post-operative analgesics [35,36], as well as chemotherapeutics [37]. By utilizing an intrathecal, rather than a trans-blood brain barrier route for contrast delivery, the difficulties associated with therapeutic agent delivery into the CNS [38] can be circumvented.

## Competing interests

The authors declare that they have no competing interests.

## Author contributions

LY carried out experiments, analyzed results, and prepared manuscript. BTK carried out experiments, analyzed results and prepared manuscript. HJW carried out experiments and analyzed results. MT carried out experiments and analyzed results. BW, RD and HB helped develop experimental concept and provided editorial assistance with manuscript. JJI, MN conceived of study concept, supervised research and prepared manuscript. All authors read and approved the final manuscript.
